# Defect-based scenario simulation teaching in the specialized skills training of nurse anesthetists: a before–after within-subject design

**DOI:** 10.1186/s12909-026-09098-7

**Published:** 2026-04-01

**Authors:** Yanli Ma, Han Li, Huan Zhang, Fengli Gao, Xiaobei Ma

**Affiliations:** 1 Department of Anesthesiology, Beijing Tsinghua Changgung Hospital, School of Clinical Medicine, Tsinghua Medicine, Tsinghua University, No. 168 Litang Road, Changping District, Beijing, 102218 China; 2 Nursing Department, Beijing Tsinghua Changgung Hospital, School of Clinical Medicine, Tsinghua Medicine, Tsinghua University, No. 168 Litang Road, Changping District, Beijing, 102218 China

**Keywords:** Nurse anesthetists, Defect, Scenario simulation

## Abstract

**Background:**

The scope of anesthesia services has been continuously extended and expanded. Nurse anesthetists (NAs) are important members of the anesthesia team, whose professional quality and practical skills are directly related to anesthesia safety and patient prognosis. How to enhance the theoretical and practical levels of NAs through scientific and systematic specialized skills training has become an urgent problem to be solved. We aimed to explore the application effect of the Defect-Based Scenario Simulation (DBSS) teaching method in specialized skills training of NAs.

**Methods:**

From February to July 2025, a total of 43 NAs were selected by cluster sampling to participate in training of traditional teaching and DBSS teaching respectively. After the training, we assessed relevant theoretical knowledge and practical specialized skills, critical thinking levels, and teaching satisfaction, as well as the quality of clinical practice.

**Results:**

Compared with traditional teaching, the DBSS teaching was associated with improvements in specialized skills levels (theoretical knowledge and practical ability), critical thinking levels, and teaching satisfaction among NAs, as well as an apparent reduction in error rates in clinical practice.

**Conclusions:**

The DBSS teaching approach may have the potential to enhance the specialized skills of NAs and contribute to anesthesia nursing quality. Therefore, it merits consideration as a training strategy for anesthesia nursing specialists.

**Supplementary Information:**

The online version contains supplementary material available at 10.1186/s12909-026-09098-7.

## Background

In contemporary health care systems, the sustained growth in surgical procedures and patients’ increasing demand for comfort-oriented care have greatly expanded the scope of anesthesiology [1]. However, against the backdrop of a relative shortage of anesthesiologists and inadequate human resource allocation, anesthetic nursing, which is a specialized field integrating anesthesiology and nursing, has become increasingly important in providing comprehensive perioperative care for patients [2, 3].

In China, the position of Nurse anesthetists (NAs) was established relatively late, with initial responsibilities concentrating on patient care in the post-anesthesia care unit [4]. To meet the evolving demands of the health care system, the role of NAs has expanded progressively to encompass multiple stages of patient care, including preoperative visits, intraoperative vital sign monitoring, as well as pain assessment and intervention, reflecting a trend toward functional diversification [5]. However, it is important to note that in China, anesthesia nursing is primarily established as a specialization development direction rather than an advanced practice role [6]. The workforce primarily comprises registered nurses transitioning from general roles, who typically lack systematic anesthesia-specific education prior to employment [7]. Employment requirements are limited to passing the National Nurse Practitioners Qualification Exam and fulfilling institution-specific training criteria, which falls substantially short of the continuing education benchmarks set by the International Federation of Nurse Anesthetists (IFNA) [8]. This training model consequently creates gaps in the specialized knowledge base and clinical skills in anesthesiology of NAs [9].

The current mainstream specialized skills training for NAs in China still predominantly follows the traditional model of “theoretical lectures plus practical demonstrations” [10–12]. This approach has a strong “lecture-based” character, placing trainees in a passive receptive state and depriving them of opportunities for active critical thinking and clinical decision-making practice [13]. Consequently, the effectiveness of translating theory into practice remains suboptimal [14, 15]. The Defect-Based Scenario Simulation (DBSS) is an instructional method defined by the use of simulation technology to create highly realistic, immersive clinical environments and standardized patients that replicate real-world practice. Through active participation in these scenarios, learners achieve acquisition of knowledge and enhancement of clinical competencies [16, 17]. The DBSS teaching approach involves the deliberate embedding of procedural flaws or latent risks within high-fidelity clinical simulations. By confronting these challenges, learners are compelled to identify, analyze, and resolve problems, thereby enhancing their risk prevention awareness and honing their clinical judgment [18, 19]. In recent years, the DBSS has demonstrated significant efficacy across various medical education domains, including training for operating room nurses [20] and neonatal resuscitation [21], underscoring its considerable pedagogical value. However, the systematic implementation of DBSS within specialized anesthesia nursing training remains a notable gap, representing a critical and promising area for future exploration.

In this study, we aimed to introduce the DBSS method into anesthesia nursing training and investigate its efficacy in enhancing the specialized skills of NAs, with the goal of generating evidence to inform the optimization of training paradigms.

## Methodology

### Design

This study followed a before–after within-subject design. All participating NAs received training in two sequential phases between February and July 2025. The traditional teaching (TT) model from February to March, followed by DBSS from June to July, with subsequent relevant assessments conducted. This study was granted an exemption from ethical approval by the Ethics Committee of Beijing Tsinghua Changgung Hospital (No: 25268-6-01). Written informed consent was obtained from all participants after they had been fully informed of the study’s purpose and implementation procedures.

### Participants

Participants comprised practicing NAs recruited from the Department of Anesthesiology at our hospital, using a cluster sampling method. The inclusion criteria were as follows: (1) holding a valid national nursing practitioner license; (2) minimum 1 year of work experience in anesthesia nursing; and (3) voluntary consent to participate. The exclusion criteria comprised individuals who withdrew during the study period and those who were not permanent staff members of the hospital.

### Curriculum development and scenario design

Although previous surveys have identified endotracheal intubation, arterial catheterization, and endotracheal extubation as the most common procedures performed by Chinese NAs [22], the *Anesthesia Nursing Manual* [23] prohibits nurses from performing endotracheal intubation and extubation independently. Consequently, the researchers ultimately selected three core techniques for standardized training and assessment: arterial catheterization, endotracheal intubation assistance, and spinal anesthesia assistance. The training curriculum encompassed both theoretical instruction and practical skill drills. The curriculum was designed to improve procedural competency in standardized protocols and foster adaptive clinical response capabilities among NAs.

**Fig. 1:**
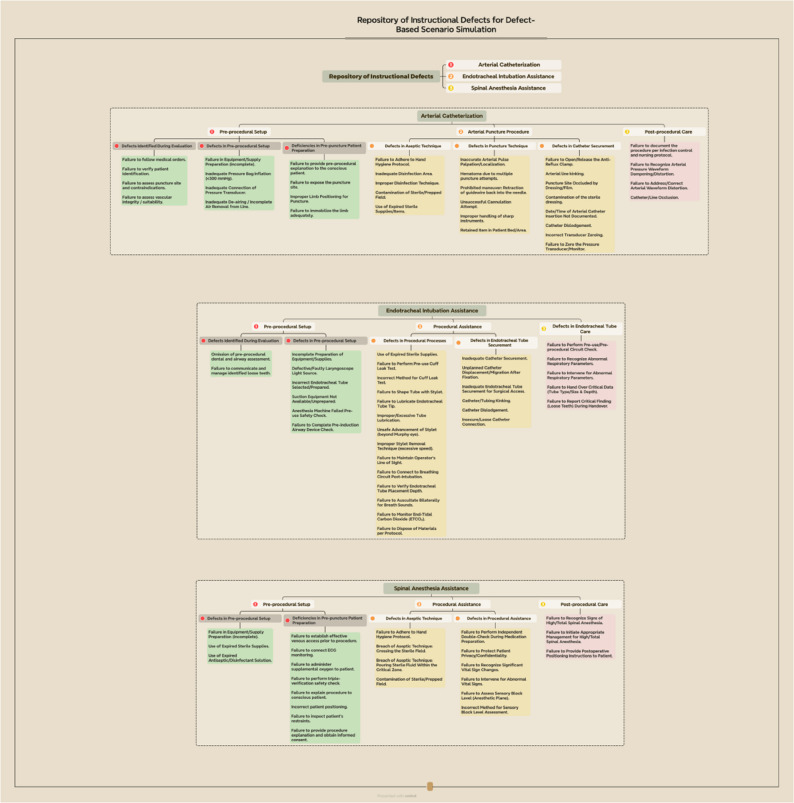
Repository of instructional defects for defect-based scenario simulation

All educational materials (Appendix 1) for this study were developed by the scenario development team. This seven-member multidisciplinary team included one N1-level NA, two faculty members, one assistant head nurse, one head nurse, and two associate chief physicians (average experience: 13 years). Their development tasks encompassed: ① standardized instructional content; ② procedural demonstration protocols; and ③ the “Repository of Instructional Defects for Defect-Based Scenario Simulation” (Fig. 1, This repository was developed exclusively for instructional design during the curriculum development phase and was not intended to serve as an outcome evaluation instrument), developed based on the learning objectives and standard operating procedures in *the Anesthesia Nursing Manual* [23] and typical errors in daily practice, involving the design of representative defect-based scripts by integrating real clinical cases and teaching goals, which were ultimately produced as micro-videos for the subsequent scenario teaching phase.

### Intervention implementation

Training was delivered by two lead instructors (N3-level senior nurse practitioners with average 17.5 years of clinical and 12.5 years of teaching experience). They were responsible for delivering lectures, demonstrating procedures, and facilitating the simulation training.

#### Phase I: Traditional teaching (TT) Model (February–March)

During February and March 2025, participants received training following the TT model. This training was structured around one core skill per week, completed over 3 weeks. Each session included: (1) a 40-minute unified lecture in which two instructors delivered systematic theoretical and operational content; (2) a 30-minute collective demonstration during which instructors demonstrated the procedures on medical manikins; and (3) a 60-minute guided hands-on practice in which NAs performed the procedures approximately 3 times on medical manikins based on the training key points (specifically: strict aseptic technique, pre-procedure preparation and assessment, standard procedural sequencing, effective communication, humanistic care, health education, and quality of implementation) under instructor observation and correction, followed by a summary and feedback on the performance.

#### Phase II: Defect-based scenario simulation (DBSS) Model (June–July)

This phase was also structured around one core skill per week and was completed over a 3-week period. The specific implementation protocol was as follows: (1) a 40-minute unified lecture in which two instructors delivered systematic theoretical and operational content; (2) viewing of a 15-minute collective video in which participants watched instructional videos featuring embedded defects across various surgical anesthesia scenarios; (3) a 30-minute group discussion where trainees identified “defect points” and documented them via an online survey platform (Wenjuanxing) using fill-in-the-blank digital forms to submit the group records; (4) a 40-minute instructor-led debriefing structured around the Gather-Analyze-Summarize (GAS) framework [24], in which the instructor utilized these submitted group records as the ‘Gather’ element, guided an ‘Analysis’ of overlooked issues, and concluded with a ‘Summary’ of standard operating procedures to eliminate misconceptions; (5) a 60-minute guided hands-on practice in which NAs performed procedures approximately 3 times on medical manikins, with instructor observation and correction, followed by a performance summary.

### Outcome assessment

To minimize evaluation bias, all assessments were conducted by two N3-level NAs (average experience: 13.0 years) not involved in routine teaching. The comprehensive evaluation comprised three components: (1) Practical skills, evaluated using structured checklists (provided in Appendix2-A), yielding a percentage score standardized to a 0–100 point scale; (2) Theoretical knowledge, assessed using two sets of written test papers (range: 0–30 points); and (3) Clinical practice quality, assessed post-training using a self-developed “Nursing Core Skills Clinical Checklist” (error rates calculated, see Appendix 2-C), which was developed independently from the instructional defect repository to comprehensively evaluate clinical skill performance). Both evaluators independently calibrated their scoring against these criteria prior to the evaluation.

### Quality control

A 2-month washout period was implemented between the traditional instruction phase and the subsequent defect-based simulation phase. Operationally, this interval was intended to prevent training fatigue and allow participants to consolidate their basic skills in routine clinical practice before advancing to complex simulations. However, that given the fixed sequential design (TT always preceded DBSS), potential order effects or time-related confounding cannot be excluded and should be acknowledged as a limitation of this study.

### Indicators

#### Primary outcome

The key outcome indicator was the core skills in anesthesia nursing, yielding a total possible score of 130 points. This composite score was derived from two components: Theoretical component (0–30 points): Assessed using two tests of comparable difficulty. Psychometric analyses were conducted separately for each test form using data from the corresponding cohort. Internal consistency reliability was calculated using the Kuder–Richardson Formula 20 (KR-20). Item discrimination was evaluated using the point-biserial correlation coefficient, and item difficulty was calculated as the proportion of correct responses (P-value). The KR-20 values were 0.85 and 0.87 for the TT and DBSS examinations [25], respectively. Item discrimination ranged from 0.20 to 0.30, and item difficulty ranged from 0.72 to 0.80 for both test forms, indicating comparable measurement properties across time points. Practical component (standardized from percentage scores to a 0–100 point scale): Assessed using the structured checklist provided in Appendix 2-A. As detailed in the checklist, the scoring was weighted across four dimensions: procedural technique (70%), communication skills (10%), humanistic care (10%), and health education (10%). For the practical exam, each trainee was randomly assigned one of the three core skills via a lottery. Assessments were administered following the completion of each training phase, initiated at approximately week 3 post-training and completed within a 14 ± 2 days window.

#### Secondary outcomes

##### Critical thinking skills

The critical thinking disposition of NAs was assessed using the Chinese version of the California Critical Thinking Disposition Inventory (CCTDI-CV), which was translated and validated by Peng et al. [26]. The scale comprises 70 items across seven dimensions: truth-seeking, open-mindedness, analyticity, systematicity, critical thinking self-confidence, inquisitiveness, and cognitive maturity. Table 1 provides brief definitions of these seven attributes.

**Table 1:** Definitions of seven attributes of CT disposition as conceptualized on the CTDI-CV

Subscale	Definition
Truth-seeking	The habit of desiring the best possible understanding of any given situation; it is following reasons and evidence where ever they may lead, even if they lead one to question cherished beliefs.
Open-mindedness	The tendency to allow others to voice views with which one may not agree. Open-minded people act with tolerance toward the opinions of others.
Critical thinking self-confidence	The tendency to trust the use of reason and reflective thinking to solve problems.
Systematicity	The tendency or habit of striving to approach problems in a disciplined, orderly, and systematic way.
Inquisitiveness	The tendency to be intellectually curious and eager to acquire new knowledge.
Analyticity	The tendency to be alert to what happens next. This is the habit of striving to anticipate both the good and the bad potential consequences or outcomes.
Cognitive maturity	The tendency to see problems as complex, rather than black and white. It is the habit of making a judgment in a timely way, not prematurely, and not with undue delay.

Responses were collected using a 6-point Likert scale, ranging from 1 (strongly agree) to 6 (strongly disagree). The total score ranges from 70 to 420, with the following interpretative categories: < 210, negative disposition; 210–279, moderate disposition; 280–349, positive disposition; and ≥ 350, strong positive disposition. The scale has been shown to have good psychometric properties, with a content validity index of 0.89 and Cronbach’s α coefficient of 0.90 [26], establishing it as the most widely used tool for measuring critical thinking disposition in China. The survey was administered using the online platform 1 week after the completion of each training model.

##### Quality of clinical practice

The scenario development team independently developed the “Clinical Checklist for Core Nursing Skills (see Appendix 2-C)” in compliance with the structured checklists. Items failing to meet predefined quality standards were recorded for error rates calculation. Clinical evaluations were conducted 3 weeks after the completion of each training phase (TT and DBSS). During the TT phase, all eligible cases performed by participating NAs within the observation period were consecutively included. The number of evaluated cases in each skill category was recorded. During the subsequent DBSS phase, case collection continued until an equivalent number of procedures was reached for each skill category to ensure comparability between training conditions. A hybrid assessment model was employed by the two N3-level NAs, combining unobtrusive direct live observation with retrospective review of operating room surveillance footage. Direct live observation was used for critical items requiring tactile feedback or close bedside inspection, including the Allen’s test (assessment of color return), airway assessment (mouth opening and neck mobility), and auscultation of breath sounds. Video review was used to evaluate broader procedural sequences, such as aseptic technique, equipment preparation, workflow coordination, and waste disposal practices. Observations covered the entire perioperative process for each procedure, defined as the period from the patient’s entry into the operating room until completion of the specific anesthesia procedure. Each checklist item was operationalized as a binary outcome (“Standard Met” or “Standard Not Met”) based on predefined criteria. Errors was recorded if an item failed to meet the standard during either live observation or video review. The number of evaluated cases differed across procedure types because procedural frequency varied in routine clinical practice. However, for each procedure category, the same number of cases was evaluated in both training phases to ensure balanced comparison. The unit of analysis was the individual clinical case. As participating NAs could perform multiple procedures during the observation period, some nurses contributed more than one observation. However, to maintain a non-punitive audit environment during both live observations and video reviews, specific nurse identifiers were not recorded. Consequently, it was not feasible to employ a clustered statistical approach, such as generalized estimating equations (GEE) or mixed-effects models. Statistical comparisons were conducted at the case level using χ² tests. Clustering by nurse was not modelled and is acknowledged as a methodological limitation.

##### Teaching satisfaction

A self-designed satisfaction questionnaire was used to subjectively evaluate the two training models (The items were generated based on the study objectives and the literature [27]). The instrument comprised 8 items rated on a 5-point Likert scale (1 = “strongly disagree” to 5 = “strongly agree”), covering dimensions such as overall satisfaction, course content, training environment, theory–practice integration, knowledge acquisition, and learning engagement. Higher total scores reflected a greater level of satisfaction. Two stand-alone questions specific to DBSS teaching were added to evaluate its perceived value and instructional design rationale. The content validity was not evaluated in this study, and the internal consistency was calculated as 0.98. Consequently, the results derived from it should be interpreted as exploratory and descriptive in nature, indicating trends rather than definitive conclusions. This electronic questionnaire was administered 1 week after each training phase.

### Statistical analysis

Normality of continuous variables was assessed using the Kolmogorov–Smirnov test. Data are presented as mean ± standard deviation for normally distributed variables or median (interquartile range) for non-normally distributed variables; categorical variables are reported as count and percentage. To align with the within-subject design, comparisons of continuous outcomes between the TT and DBSS phases were performed using the Paired Samples t-test for normally distributed data and the Wilcoxon signed-rank test for non-normally distributed data. Mean differences (change scores) were calculated and reported with 95% confidence intervals (CIs). Categorical data comparisons were conducted using the χ2 test. The distribution of the three practical skills between the TT and DBSS phases was compared using the chi-square test to assess whether the lottery assignment resulted in comparable skill distributions across the two assessment time points. All analyses were performed with IBM SPSS Statistics 26.0 (IBM Corp., Armonk, NY, USA), with a significance threshold of *P* < 0.05.

## Results

**Fig. 2:**
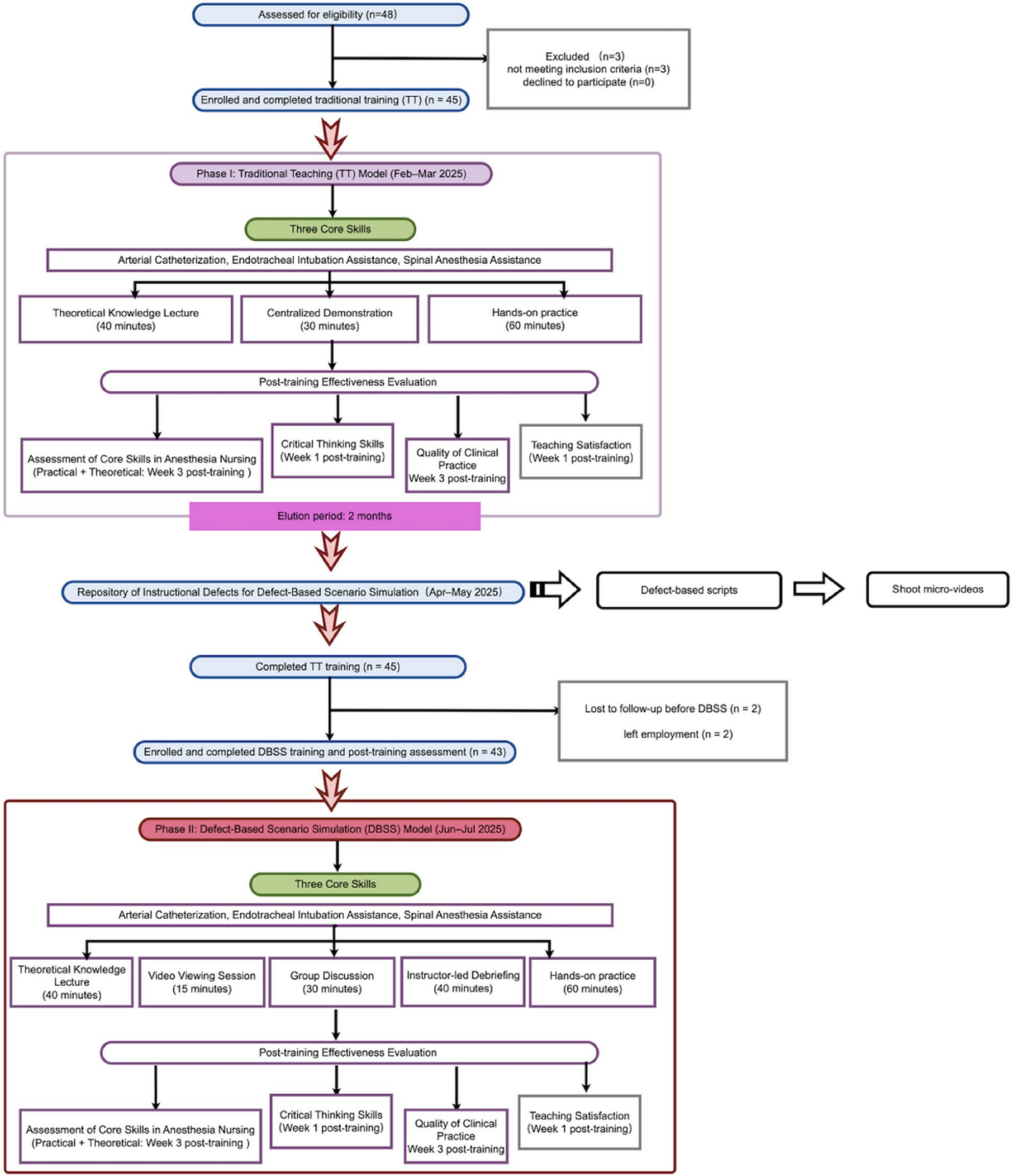
The research flowchart

A total of 48 NAs were initially included in the study. After excluding three NAs who were on maternity leave, 45 NAs received TT. Subsequently, two NAs left the study before the start of DBSS, leaving 43 NAs who completed the entire research process. The research flowchart is shown in Fig. 2.

The final study population included 10 men and 33 women, with average age 27 ± 4.63 years. Detailed demographic data are presented in Table 2.

**Table 2:** General information of research participants (*N* = 43)

Variable	Categories	*n* (%)
Sex	Male	10 (23.3)
	Female	33 (76.7)
Age (mean age, 27.77 ± 4.63 y)		
Average year of work (5.72 ± 3.98 y)		
Years’ working experience in anesthesia nursing	1–3	21 (48.9)
	4–6	9 (20.9)
	> 6	13 (30.2)
Education level	Bachelor’s	42 (97.6)
	Graduate	1 (2.3)
Clinical title	Registered nurse (RN)	15 (34.9)
	Nurse practitioner	21 (48.8)
	Nurse-in-charge	7 (16.3)
Professional level	N1	22 (51.2)
	N2	12 (27.9)
	N3	9 (20.9)

Primary outcome as follows: The lottery assignment resulted in a distribution of the three assessed procedures between the TT and DBSS phases (TT phase: arterial catheterization *n* = 16 (37.2%), endotracheal intubation assistance *n* = 13 (30.2%), spinal anesthesia assistance *n* = 14 (32.6%); DBSS phase: arterial catheterization *n* = 17 (39.5%), endotracheal intubation assistance *n* = 12 (27.9%), spinal anesthesia assistance *n* = 14 (32.6%). A chi-square test revealed no significant difference in the distribution of skills between the two phases (χ² = 0.089, *P* = 0.956), indicating that the lottery assignment successfully balanced the types of procedures assessed at each time point.

**Fig. 3:**
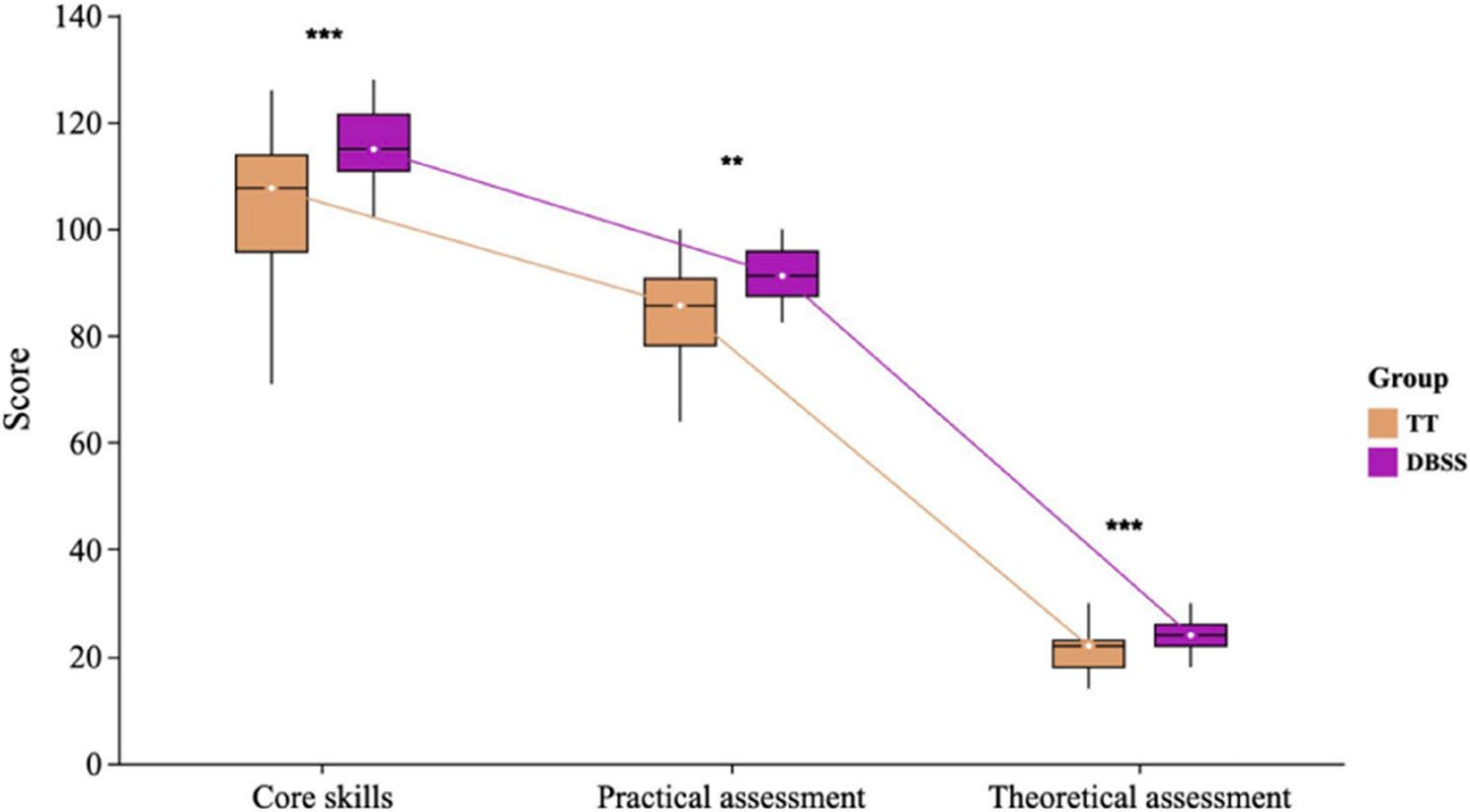
Comparison of scores in anesthesia nursing between the two instructional models. (TT: traditional teaching, DBSS: Defect-Based Scenario Simulation. *P < 0.05, **P < 0.001, ***P < 0.0001)

In this study, Theoretical scores rose from 20.9 ± 3.89 to 23.7 ± 2.96 (*P* < 0.01), and practical scores increased from 83.8 ± 12.78 to 91.4 ± 10.22 (*P* < 0.01). The DBSS model significantly improved the NAs’ core skill scores (115.1 ± 11.35 vs. 104.7 ± 14.59, *P* < 0.01) compared with TT (Fig. 3).

**Fig. 4:**
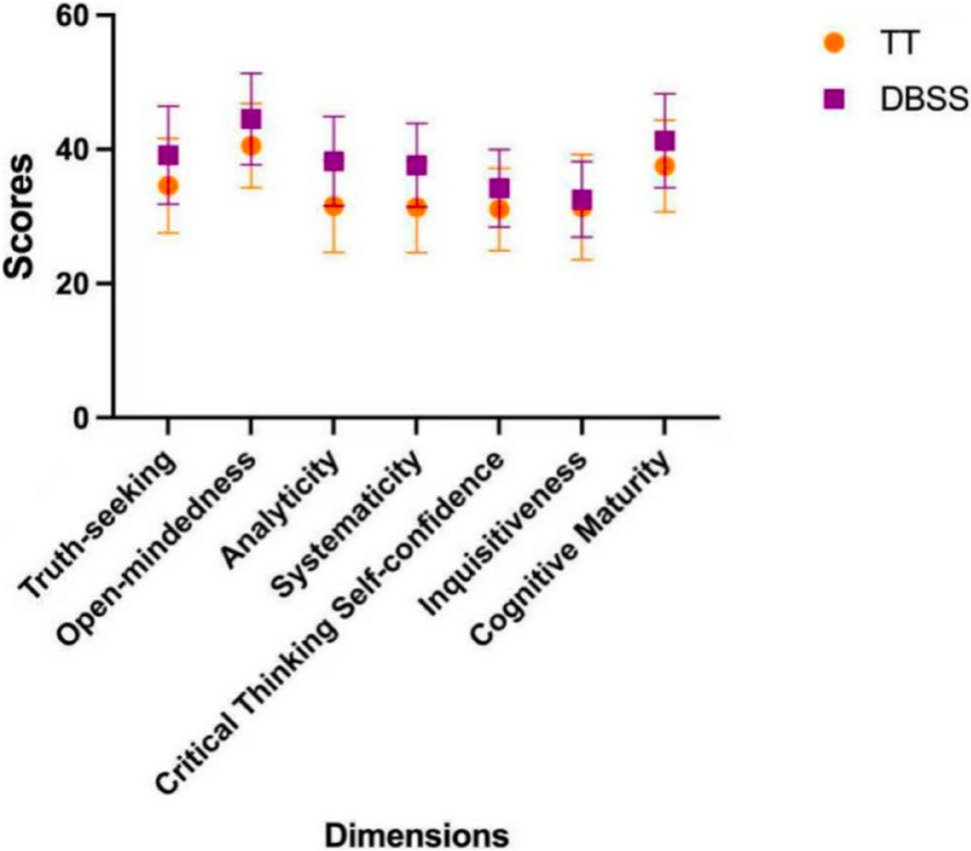
Comparison of critical thinking between the two instructional models. (TT: traditional teaching, DBSS: Defect-Based Scenario Simulation)

The secondary outcome measures are as follows: NAs demonstrated statistically significant improvement in critical thinking skills after DBSS (238.01 ± 22.72 vs. 267.53 ± 20.96, *P* < 0.001). The results showed that both training models led to moderate levels of critical thinking among NAs before and after the program. The highest scores were recorded for “open-mindedness” and “cognitive maturity” indicating participants’ willingness to objectively consider new evidence and perspectives while maintaining prudence and judgment in decision-making. All items except the “Inquisitiveness” item showed significant improvements (Inquisitiveness: 31.36 ± 7.84 vs. 32.55 ± 5.64, *P* > 0.05; Truth-seeking: 34.58 ± 7.06 vs. 39.12 ± 7.31, *P* < 0.05; Open-mindedness: 40.55 ± 6.27 vs. 44.52 ± 6.81, *P* < 0.05; Analyticity: 31.55 ± 6.86 vs. 38.21 ± 6.64, *P* < 0.001; Systematicity: 31.39 ± 6.77 vs. 37.64 ± 6.23, *P* < 0.001; Critical thinking self-confidence: 31.07 ± 6.12 vs. 34.21 ± 5.78, *P* < 0.05;Cognitive maturity: 37.51 ± 6.82 vs. 41.28 ± 7.00, *P* < 0.05) (Fig. 4).

**Fig. 5:**
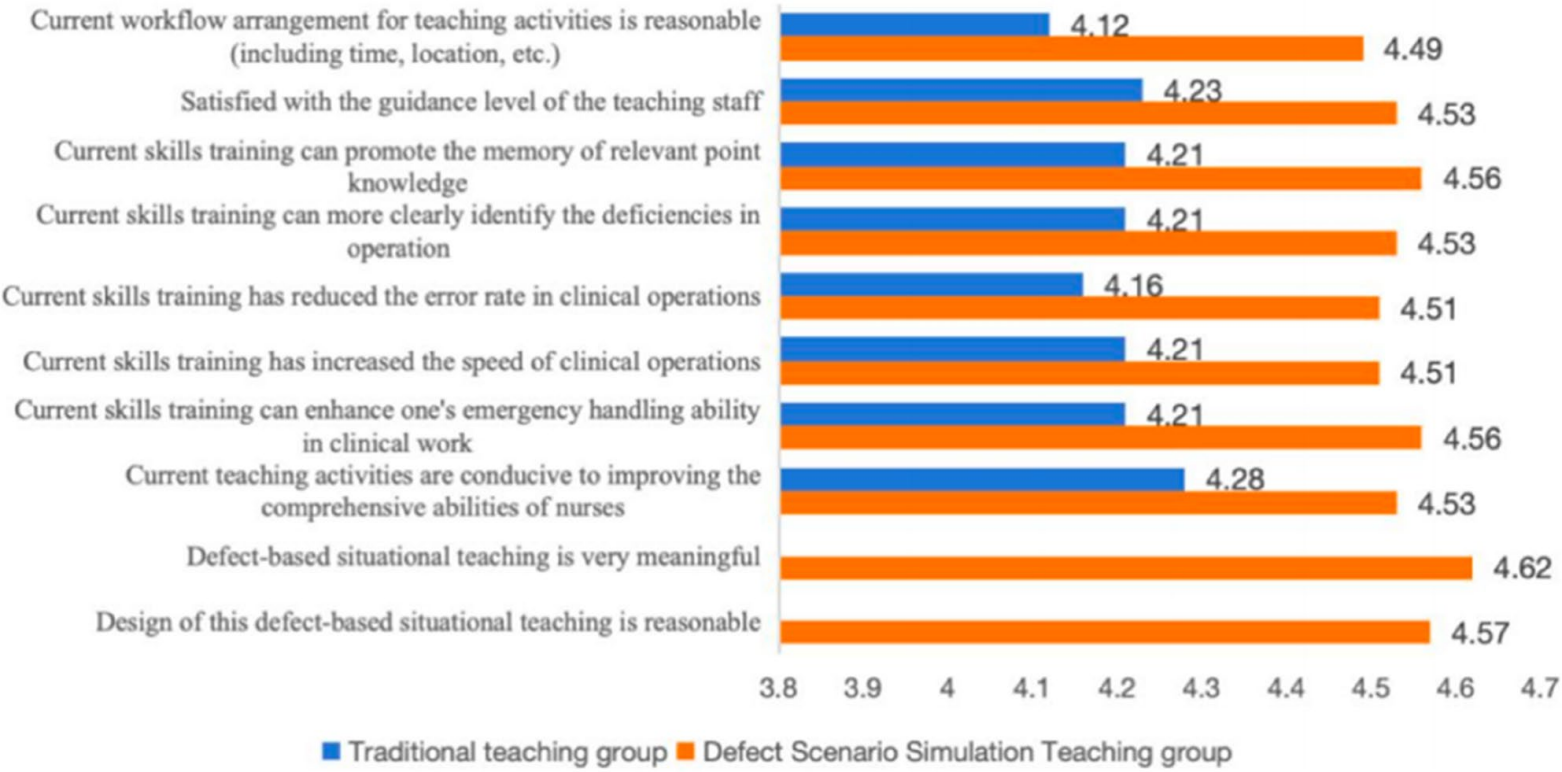
Comparison of satisfaction Scores under the two Instructional models. (TT: traditional teaching, DBSS: Defect-Based Scenario Simulation)

The NAs’ scores for satisfaction for DBSS (36.23 ± 4.37) were significantly higher than those for TT (33.63 ± 4.98), with a statistically significant difference (paired t-test, *P* < 0.05). In a dedicated survey on this teaching model, participants gave the item “I find the defect-based situational teaching highly meaningful” a score of 4.56 ± 0.55 and “I believe the design of defect-based situational teaching is reasonable” a score of 4.53 ± 0.55, indicating widespread recognition of the DBSS’s value and design (Fig. 5).

The results of the clinical practice quality assessments: arterial catheterization (291 cases for each instructional model), endotracheal intubation assistance (229 cases for each instructional model), and spinal anesthesia assistance (96 cases for each instructional model). The three most error-prone procedures are listed in the Table 3 (complete content refer to “Supplementary Table 3”). Following defect-based situational teaching, error rates in these core skills showed significant reduction .

**Table 3:** Error rates in clinical practice quality audits

Item	Problem points of verification	Occurrence count (error rate %)		χ^2^ test
		TT	DBSS	
Arterial catheterization	1. Allen test not performed	259 (89)	76 (26)	< 0.001
	2. Inadequate disinfection area	135 (46)	11 (4)	< 0.001
	3. Inadequate limb immobilization	87 (30)	10 (3)	< 0.001
Endotracheal intubation assistance	1. Failure to monitor end-tidal carbon dioxide (ETCO_2_)	178 (78)	13 (6)	< 0.001
	2. Pre-induction preparation omissions: Failure to confirm endotracheal tube size Endotracheal tube not unpacked and ready for use	132 (58)	19 (8)	< 0.001
	3. Airway assessment not performed	125 (55)	17 (7)	< 0.001
Spinal anesthesia assistance	1. Failure to check expiry dates of sterile supplies	24 (25)	7 (7)	< 0.01
	2. Failure to instantly commence blood pressure monitoring and active management following successful puncture	17 (18)	7 (7)	< 0.05
	3. Improper technique for sensory block level assessment	16 (17)	4 (2)	< 0.01

## Discussion

This study observed an increase in core skill scores among NAs following the DBSS phase, compared to scores after the preceding TT phase. Significant improvements were noted in both theoretical and practical components. These sequential gains suggest that the DBSS teaching model may be associated with enhanced knowledge and skill outcomes, a trend that aligns with previous findings [13, 28, 29]. The possible mechanism is as follows. First, the introduction of “defects” can stimulate learning motivation and cognitive conflict, thereby enhancing the understanding of theoretical knowledge. Foreign studies have confirmed that error-based learning strategies can improve learners’ metacognitive monitoring and long-term memory retention [30, 31]. In this study, trainees engaged in repeated discussions during the debriefing about “the causes of errors and the rationale for correct procedures” thereby deepening their structured understanding of theoretical knowledge. Second, exposing errors in a safe simulated environment and correcting them promptly facilitates skill formation and consolidation. Through the cyclical process of “operation–error exposure–correction–re-practice”, DBSS enables trainees to reinforce correct movement patterns and eliminate misunderstanding through repeated practice. As noted by Gaba [32], the value of scenario-based simulation teaching lies not only in replicating correct operations but also in providing a safe “error-prone environment” that enables learners to learn from mistakes and grow through reflection. Therefore, the participants in this study were practicing NAs with a minimum of one year of anesthesia nursing experience, rather than nursing students. In contrast, the outcomes might differ if the intervention were applied to anesthesia nursing students. Students are typically at the “novice” or “advanced beginner” stage and may lack sufficient clinical context to rapidly recognize subtle procedural defects.

As a critical component of the anesthesia team, the professional competence and technical proficiency of NAs may affect both surgical safety and patient outcomes [33, 34]. Current training for anesthesia nurses predominantly uses TT models centered on instructor-led lectures and hands-on demonstrations [10–12]. While systematic in knowledge delivery, this approach falls short in terms of fostering deep understanding and skills transfer. The DBSS teaching model adopted in this study represents a substantial innovation in pedagogical philosophy and learning mechanisms [35]. This kind of situational learning with defects as carriers promotes the integration of theory, skills, and reflection, which is in line with Kolb’s Learning Cycle Model of “experience–reflection–conceptualization–application” [36], encouraging trainees to proactively identify issues, analyze root causes, and devise improvement strategies, thereby enabling them to gain profound learning experiences in realistic, stress−controlled scenarios [37]. Therefore, the DBSS model may be more effective than the traditional model in achieving the internalization of theoretical knowledge and the transfer of operational skills.

The results of this study indicated that critical thinking ability scores among NAs were significantly higher after the DBSS intervention. This finding suggests that DBSS training might be able to enhance the critical thinking ability of NAs, aligns with findings reported in both domestic and international literature [38, 39]. However, the critical thinking ability of the NAs in both groups after training was less than 280 points, indicating that the nurses generally did not have positive critical thinking ability. This might be related to the cramming education method, suggesting that the nursing education model needs innovation. In the increasingly complex health care system, nurses require not only solid professional knowledge and practical skills but also the ability to conduct systematic analysis, identify risks, and make clinical decisions to address ever-changing clinical scenarios and potential hazards [40, 41]. Critical thinking is recognized as a core competency for safe and effective clinical nursing practice [42, 43].

As key personnel in high-risk anesthesia procedures, nurses’ critical thinking directly affects both surgical safety and efficiency of team collaboration [44]. Whereas conventional training models emphasize standardized protocols and correct answers, thereby demonstrating mastery of technical skills, they often fail to develop trainees’ ability to identify issues and devise innovative solutions in complex scenarios [45]. The DBSS teaching approach addresses this limitation in its design concept. This method intentionally includes “defects” or “errors” within simulated cases, which may arise from equipment failure, drug confusion, sudden changes in disease status, communication obstacles, or handover errors, among others [46]. This “active problem exposure” instructional design enables trainees to identify issues, analyze causes, assess risks, and make decisions in highly realistic clinical settings, thereby activating higher-order cognitive processes [18, 19]. Although DBSS currently lacks an independent theoretical framework, its pedagogical philosophy aligns closely with multiple established educational theories [47, 48]. As the Reflective Learning Theory, through identifying errors, analyzing biases, discussing causes, and developing improvement strategies, learners undergo a cognitive shift from passive execution to active thinking, achieving deep learning and restructuring of cognitive patterns [49]. Research demonstrates that this reflective learning mechanism can significantly enhance nurses’ abilities in clinical decision-making, including logical reasoning, risk anticipation, and ethical judgment [50, 51]. International studies have also confirmed that “error-based learning” effectively fosters critical thinking and improves clinical decision-making capabilities [52].

In this study, we compared the NAs’ actual clinical performance of the two training models. The results showed that the highest error rates observed among trainees for the three core skills was lower in the DBSS phase than in the preceding TT phase. This finding suggests that the learning from the DBSS model was associated with a measurable reduction in performance errors, providing support for its potential to facilitate the transfer of learning to practice. Further studies are needed to confirm this transfer in actual clinical settings. Barsuk demonstrated that a simulation-based mastery learning program enhanced residents’ skills in central venous catheter insertion and reduced related complications when providing actual patient care [53]. Learning transfer is a key objective of higher-order cognitive learning. According to Perkins and Salomon’s Transfer of Learning Theory, the transfer of knowledge or skills from the classroom to practice depends on “situational similarity” and “depth of processing” during the learning process [54]. The TT method, which lack authentic contexts and contingency intervention designs, often confine learners to rote memorization and procedural imitation, making it difficult to flexibly apply acquired skills in complex clinical environments [55]. By contrast, DBSS teaching substantially enhances situational consistency between training and real-world settings by introducing highly realistic clinical scenarios with integrated pre-designed errors [48]. Trainees can more quickly identify potential risks and adjust their behavior when facing real clinical operations, thereby reducing error rates [29].

The satisfaction scores reported after the DBSS teaching model were higher than those after the TT model, which suggests that NAs may prefer the DBSS teaching model. In the single-item survey, the items “I find DBSS highly meaningful” and “I believe the design of DBSS is reasonable” both received high scores, indicating widespread recognition among NAs regarding the value and rationality of the DBSS model. The existing literature indicates that the DBSS method creates authentic clinical tension and challenges by intentionally introducing “errors” “sudden situations” or “system defects” in the teaching context. This approach enables learners to experience “cognitive engagement” and “emotional resonance” during problem-solving processes, thereby enhancing their learning motivation and sense of achievement [13]. This highly immersive and task-oriented teaching experience has been shown to considerably enhance learning satisfaction and retention. From the perspective of instructional psychology, DBSS enables learners to achieve deep understanding through active exploration and reflective construction [21]. Unlike traditional teacher-centered instruction, this approach emphasizes learners’ agency and practical engagement [56]. The DBSS method allows NAs to experience enhanced self-efficacy and professional growth during the learning process, thereby greatly improving their satisfaction with the teaching model [20].

### Limitations

This study has several limitations. First, the before-and-after design without a parallel control group introduces potential time- and order-related confounding. Because TT was consistently implemented prior to DBSS, improvements observed during the DBSS phase may partially reflect maturation, increasing clinical experience, repeated testing effects, or secular trends rather than the intervention alone. Although an interval was incorporated between phases to prevent training fatigue, it cannot eliminate cumulative learning effects and may itself introduce additional time-related bias.

Second, Our initial study design aimed to minimize selection bias precisely through the lottery method. Each participant was randomly assigned to one of three practical skills at each assessment point. Our intention was to treat the entire cohort of NAs (*n* = 43) as a single representative sample. By averaging the scores across all three skills, we sought to obtain a global estimate of the cohort’s practical skill at each phase (TT and DBSS). Although we confirmed that the distribution of practical skills tested was comparable between the TT and DBSS phases (*P* > 0.05), we cannot entirely rule out the possibility that inherent differences in skill difficulty may have influenced the overall practical assessment scores. Future studies with larger sample sizes should consider assessing all participants on the same skill or employing a crossover design to better control for this potential confounding.

Third, clinical performance was analyzed at the case level. As participating NAs could contribute multiple observed procedures during each phase, observations were not statistically independent at the nurse level. To maintain a non-punitive audit environment during the observations, specific nurse identifiers were intentionally not recorded. Consequently, clustering by individual nurse could not be explicitly modeled in the analysis, which may have resulted in underestimation of standard errors and inflation of statistical significance.

Fourth, participants were recruited from a single regional hospital with a relatively limited sample size, which may constrain external validity. Multi-center randomized controlled studies with appropriate statistical modelling of repeated measures are warranted to confirm these findings.

Finally, the flawed scenarios designed for DBSS primarily focused on common technical and communication deficiencies and did not systematically incorporate highly complex clinical situations or interprofessional team dynamics. Future studies should expand scenario diversity and evaluate longer-term educational and patient-safety outcomes.

## Conclusions

The results of this study suggest an association between the DBSS teaching and observed improvements in the core skills and critical thinking ability of NAs. This overall positive trend may extend to potentially benefit the quality of clinical practice. Through the purposeful simulation of typical errors and potential risk events in the teaching environment, learners can achieve transformation from passive reception of information to active exploration in the process of experiencing, identifying, and correcting errors in the simulated environment, which promotes deep understanding of knowledge and the application of skills. Meanwhile, This teaching model reported higher learning interest and satisfaction, which might help strengthen their clinical decision-making and risk management awareness. The DBSS method provides a novel, highly practical teaching strategy for anesthesia nursing specialty training, may have a vital role in improving NAs’ clinical competence and consciousness about patient safety. In the future, the long-term teaching effect should be verified in multi-center and larger sample studies, its application value at different levels of nursing education should be explored.

## Supplementary Information

Supplementary Material 1.

Supplementary Material 2.

Supplementary Material 3.

Supplementary Material 4.

Supplementary Material 5.

Supplementary Material 6.

Supplementary Material 7.

## Data Availability

The datasets used and/or analyzed during the current study are available from the corresponding author on reasonable request.

## Abbreviations

DBSS: the Defect-Based Scenario Simulation
TT: the Traditional Teaching
NAs: Nurse anesthetists
IFNA: the International Federation of Nurse Anesthetists
RN: Registered nurse
ETCO2: End-Tidal Carbon Dioxide
