# Supplementary material for: Defect-based scenario simulation teaching in the specialized skills training of nurse anesthetists: a before–after within-subject design
Source: BMC Med Educ. 2026 Apr 1;26:752. doi: 10.1186/s12909-026-09098-7 (PMC13169835; doi:10.1186/s12909-026-09098-7)
Supplement: Supplementary file 1 — Supplementary Material 1. [file 12909_2026_9098_MOESM1_ESM.docx]

Appendix 1 Clinical Checklist for Core Nursing Skills

Clinical Checklist Arterial Catheterization

Date:  Persons Checked:  Checks Completed: Inspector:

| Category | Count  Tally marks (正) were used for manual counting | Category | Count  Tally marks (正) were used for manual counting |
| --- | --- | --- | --- |
| Failure to verify patient identity |  | Use of Expired Sterile Supplies/Items |  |
| Failure to follow medical orders |  | Inaccurate Arterial Pulse Palpation/Localization |  |
| Failure to assess puncture sites and procedure-related contraindications |  | Hematoma due to Multiple Attempts/Technical Difficulty |  |
| Failure to assess arterial suitability (via Allen test) |  | Prohibited Maneuver: Retraction of guidewire back into the needle |  |
| Incomplete Preparation of Equipment/Supplies |  | Unsuccessful Cannulation Attempt |  |
| Inadequate Pressure Bag Maintenance (<300 mmHg) |  | Improper handling of sharp instruments |  |
| Inadequate Connection of Arterial Pressure Sensor |  | Retained Item in Patient Bed/Area |  |
| Inadequate De-airing / Incomplete Air Removal from Arterial Pressure Line |  | Failure to Open/Release the Anti-Reflux Clamp |  |
| Failure to provide procedure explanation and obtain informed consent |  | Puncture Site Occluded by Dressing/Film |  |
| Failure to expose the puncture site |  | Date/Time of Arterial Catheter Insertion Not Documented |  |
| Improper Limb Positioning for Puncture |  | Catheter Dislodgement |  |
| Inadequate Limb Immobilization |  | Incorrect Transducer Zeroing |  |
| Failure to Adhere to Hand Hygiene Protocol |  | Failure to Zero the Pressure Transducer/Monitor |  |
| Inadequate Disinfection Area |  | Failure to Recognize Arterial Pressure Waveform Dampening/Distortion |  |
| Improper Disinfection Technique |  | Failure to Address/Correct Arterial Waveform Distortion |  |
| Contamination of Sterile/Prepped Field |  | Catheter/Line Occlusion |  |

Clinical Checklist Endotracheal Intubation Assistance

Date:  Persons Checked:  Checks Completed: Inspector:

| Category | Count  Tally marks (正) were used for manual counting | Category | Count  Tally marks (正) were used for manual counting |
| --- | --- | --- | --- |
| Failure to verify patient identity |  | Endotracheal Tube Not Shaped with Stylet |  |
| Dental Assessment: Not Performed |  | Tube Tip Lubrication: Not Applied |  |
| Airway Assessment: Not Performed |  | Unsafe Advancement of Stylet (beyond the Murphy eye) |  |
| Failure to Notify Anesthesiologist of Loose Tooth |  | Improper Stylet Removal Technique (excessive speed) |  |
| Incomplete Preparation of Equipment/Supplies |  | Failure to Maintain a Clear Line of Sight During Stylet Removal |  |
| Video Laryngoscope Light Source (Insufficient/Broken) |  | Failure to Connect to Ventilator/Breathing Circuit Post-Intubation |  |
| Incorrect Endotracheal Tube Selection (size/type) |  | Failure to Verify Endotracheal Tube Placement Depth |  |
| Suction Device: Not Available |  | Failure to Auscultate Bilaterally for Breath Sounds |  |
| Failure to Perform Pre-operative Anesthesia Machine Check |  | Failure to Monitor End-Tidal Carbon Dioxide (ETCO₂) |  |
| Pre-induction Preparation Omissions:  Failure to confirm endotracheal tube size  Endotracheal tube not unpacked and ready for use |  | Inadequate Endotracheal Tube Securement |  |
| Use of Expired Sterile Supplies/Items |  | Unplanned Endotracheal Tube Displacement/Migration After Securement |  |
| Cuff Leak Test: Not Performed |  |  |  |

Clinical Checklist Spinal Anesthesia Assistance

Date:  Persons Checked:  Checks Completed: Inspector:

| Category | Count  Tally marks (正) were used for manual counting | Category | Count  Tally marks (正) were used for manual counting |
| --- | --- | --- | --- |
| Failure to verify patient identity |  | Incomplete Preparation of Equipment/Supplies |  |
| Venous Access: Not Established |  | Failure to Adhere to Hand Hygiene Protocol |  |
| ECG Monitoring: Not Applied |  | Violation of Aseptic Technique: Breaching/Crossing the Sterile Field |  |
| Pre-procedure Oxygen: Not Administered |  | Aseptic Technique Violation Resulting in Field Contamination |  |
| Failure to provide procedure explanation and obtain informed consent |  | Breakdown in Team Safety Check During Medication Retrieval: Independent Verification Omitted |  |
| Incorrect Positioning for Spinal Anesthesia |  | Failure to Protect Patient Privacy |  |
| Patient Restraint: Not Assessed |  | Failure to instantly commence blood pressure monitoring and active management following successful puncture. |  |
| Failure to Provide Pre-procedural Instructions for Spinal Anesthesia |  | Failure to Assess/Test the Sensory Block Level (Anesthetic Plane) |  |
| Failure to Check Expiry Dates of Sterile Supplies |  | Improper Technique for Sensory Block Level Assessment |  |
