# Supplementary material for: Defect-based scenario simulation teaching in the specialized skills training of nurse anesthetists: a before–after within-subject design
Source: BMC Med Educ. 2026 Apr 1;26:752. doi: 10.1186/s12909-026-09098-7 (PMC13169835; doi:10.1186/s12909-026-09098-7)
Supplement: Supplementary file 2 — Supplementary Material 2. [file 12909_2026_9098_MOESM2_ESM.docx]

Appendix 1 Educational materials for the study

**① Standardized Instructional Content**

| Module | Core skill | Learning objectives | Teaching format |
| --- | --- | --- | --- |
| Module 1 | Arterial Catheterization | Patient assessment; indications and contraindications for arterial catheterization; standardized procedural workflow; communication skills; humanistic care and health education; and prevention and management of abnormal conditions. | Lecture + demo + DBSS |
| Module 2 | Endotracheal Intubation Assistance | Pre-intubation examination and assessment; indications and contraindications for endotracheal intubation; standardized procedural workflow; communication skills; humanistic care and health education; and prevention and management of complications and abnormal conditions. | Lecture + demo + DBSS |
| Module 3 | Spinal Anesthesia Assistance | Pre-procedure assessment; indications and contraindications for spinal anesthesia; standardized procedural workflow; communication skills; humanistic care and health education; and prevention and management of complications and abnormal conditions. | Lecture + demo + DBSS |

**② Summary of standardized procedural demonstration protocols**

| Module | Core skill | Demonstration focus areas |
| --- | --- | --- |
| Module 1 | Arterial Catheterization | Patient assessment; aseptic technique; puncture and cannulation; waveform verification; fixation |
| Module 2 | Endotracheal Intubation Assistance | patient assessment and airway evaluation; standardized equipment preparation; correct assistance techniques during laryngoscopy and intubation; appropriate endotracheal tube fixation; continuous airway and ventilation monitoring. |
| Module 3 | Spinal Anesthesia Assistance | pre-procedure patient assessment; establishment of intravenous access and baseline vital sign monitoring prior to the procedure; strict adherence to aseptic technique; appropriate patient positioning; protection of patient privacy and dignity; post-procedure vital sign monitoring; and assessment of the level and effectiveness of neuraxial blockade. |

**③ Representative Instructional Scenarios Developed from the Repository of Instructional Defects for DBSS**

This supplementary file provides representative examples of how selected instructional defects from the repository (Figure 2) were translated into defect-based scenario scripts for teaching purposes. The examples are illustrative rather than exhaustive and are not intended for outcome evaluation or scoring.

| Module | Core skill | Procedural phase | Defect category (from Fig. 2) | Representative instructional defect | Scenario design focus | Learning objective / Prompt |
| --- | --- | --- | --- | --- | --- | --- |
| Module 1 | Arterial Catheterization | Pre-procedural Setup | Defects Identified During Evaluation | Failure to perform Allen test | Omission of Allen test prior to arterial catheterization, increasing the risk of unrecognized inadequate collateral circulation and subsequent ischemic complications. | Prompt NAs to identify contraindications to arterial catheterization, justify the need for vascular assessment (e.g., Allen test), and explain potential consequences of omission. |
|  |  |  | Defects in Pre-procedural Setup | Inadequate De-airing / Incomplete Air Removal from Line | Simulated abnormal arterial pressure waveform caused by residual air in the pressure monitoring line, requiring identification of the underlying cause. | Prompt NAs to recognize abnormal arterial waveforms, identify inadequate de-airing as the underlying cause, and perform appropriate troubleshooting to restore accurate pressure monitoring. |
|  |  |  | Deficiencies in Pre-puncture Patient Preparation | Improper Limb Positioning for Puncture. | Inaccurate anatomical localization due to suboptimal limb positioning, increasing the likelihood of failed arterial puncture and repeated attempts. | Prompt NAs to recognize the impact of improper limb positioning on anatomical landmark identification and adjust patient positioning to optimize puncture success. |
|  |  | Arterial Puncture Procedure | Defects in Aseptic Technique | Inadequate Disinfection Area | Insufficient disinfection coverage increasing the risk of catheter-related infection. | Prompt NAs to recognize inadequate disinfection practices, identify potential infection risks, and justify appropriate vascular preparation. |
|  |  |  | Defects in Puncture Technique | Hematoma due to multiple puncture attempts | Repeated puncture attempts increasing the risk of hematoma formation and neurovascular injury. | Prompt NAs to recognize factors leading to multiple puncture attempts, optimize puncture technique, and justify strategies to minimize vascular and neural injury. |
|  |  |  | Defects in Catheter Securement | Puncture Site Occluded by Dressing/Film | Prompt NAs to recognize factors leading to multiple puncture attempts, optimize puncture technique, and justify strategies to minimize vascular and neural injury. | Prompt NAs to evaluate catheter securement methods, ensure adequate visualization of the puncture site, and justify decisions that facilitate early identification of complications. |
|  |  | Post-procedural Care | / | Failure to Recognize Arterial Pressure Waveform Dampening/Distortion | Simulated dampened or distorted arterial pressure waveform resulting from line-related issues, requiring recognition and interpretation by NAs. | Prompt NAs to recognize abnormal arterial pressure waveforms, identify potential causes of waveform dampening or distortion, and initiate appropriate troubleshooting measures. |
| Module 2 | Endotracheal Intubation Assistance | Pre-procedural Setup | Defects Identified During Evaluation | Omission of pre-procedural dental and airway assessment | Failure to identify dental and airway risk factors prior to intubation, leading to increased risk of dental injury and difficult airway management. | Prompt NAs to perform a structured pre-procedural dental and airway assessment, identify potential risk factors (e.g., loose teeth, limited mouth opening, restricted neck mobility), and anticipate appropriate airway management strategies. |
|  |  |  | Defects in Pre-procedural Setup | Suction Equipment Not Available/Unprepared | Inadequate preparation and availability of suction equipment, increasing the risk of aspiration and airway compromise during intubation. | Prompt NAs to verify the availability and functionality of suction equipment before airway management and to recognize its critical role in maintaining airway patency and preventing aspiration. |
|  |  | Procedural Assistance | Defects in Procedural Processes | Failure to Auscultate Bilaterally for Breath Sounds  Failure to Monitor End-Tidal Carbon Dioxide (ETCO₂) | Failure to confirm bilateral breath sounds after intubation, increasing the risk of unrecognized tube malposition. | Prompt NAs to perform and interpret bilateral chest auscultation after intubation and to justify its role in confirming correct endotracheal tube placement. |
|  |  |  | Defects in Endotracheal Tube Securement | Unplanned Catheter Displacement/Migration After Fixation | Insufficient endotracheal tube fixation causing post-intubation tube migration and compromised airway safety. | Prompt NAs to recognize signs of endotracheal tube displacement, apply standardized fixation techniques, and justify the need for continuous airway monitoring and re-verification of tube position after fixation. |
|  |  | Defects in Endotracheal Tube Care | / | Failure to Recognize Abnormal Respiratory Parameters | Failure to identify abnormal respiratory parameters (e.g., respiratory rate, tidal volume, airway pressure, SpO₂, ETCO₂) indicating inadequate ventilation or evolving airway compromise during or after endotracheal intubation. | Prompt NAs to recognize abnormal respiratory parameters using multimodal monitoring and to interpret these changes as early warning signs of compromised ventilation or airway instability. |
| Module 3 | Spinal Anesthesia Assistance | Pre-procedural Setup | Defects in Pre-procedural Setup | Use of Expired Antiseptic/Disinfectant Solution | Use of expired or improperly stored antiseptic/disinfectant solution during skin preparation for spinal anesthesia, compromising aseptic technique and increasing the risk of neuraxial infection. | Failure to verify the validity of antiseptic/disinfectant solution used for skin preparation before spinal anesthesia, posing a risk of neuraxial infection. |
|  |  |  | Deficiencies in Pre-puncture Patient Preparation | Failure to connect ECG monitoring | Omission of ECG monitoring during spinal anesthesia assistance, limiting timely detection of hemodynamic or cardiac changes associated with sympathetic blockade or high spinal anesthesia. | Prompt NAs to recognize the necessity of continuous ECG monitoring during spinal anesthesia, ensure timely connection of monitoring equipment, and justify its role in early detection of cardiovascular complications, including high or total spinal anesthesia. |
|  |  | Procedural Assistance | Defects in Aseptic Technique | Contamination of Sterile/Prepped Field | Breakdown of sterile technique during spinal anesthesia assistance, leading to contamination of the prepped field and increased risk of neuraxial infection. | Prompt NAs to identify breaches in sterile technique, maintain strict aseptic boundaries throughout spinal anesthesia assistance, and justify the importance of sterility in preventing neuraxial infections such as meningitis or epidural abscess. |
|  |  |  | Defects in Procedural Assistance | Failure to Protect Patient Privacy/Confidentiality | Inadequate protection of patient privacy during spinal anesthesia assistance, including unnecessary exposure of the patient or failure to maintain dignity during positioning and preparation. | Prompt NAs to recognize situations in which patient privacy may be compromised, implement appropriate draping and communication strategies, and justify the importance of protecting patient dignity and confidentiality during neuraxial anesthesia procedures. |
|  |  | Post-procedural Care | / | Failure to Recognize Signs of High/Total Spinal Anesthesia | Failure to identify early or progressive signs of high or total spinal anesthesia, such as hypotension, bradycardia, respiratory distress, upper limb weakness, or altered consciousness following neuraxial anesthesia. | Prompt NAs to recognize and interpret clinical signs suggestive of high or total spinal anesthesia through continuous monitoring and patient assessment. |

For each defect category, representative instructional defects are provided as examples. The list is illustrative rather than exhaustive.
