# Supplementary material for: Defect-based scenario simulation teaching in the specialized skills training of nurse anesthetists: a before–after within-subject design
Source: BMC Med Educ. 2026 Apr 1;26:752. doi: 10.1186/s12909-026-09098-7 (PMC13169835; doi:10.1186/s12909-026-09098-7)
Supplement: Supplementary file 3 — Supplementary Material 3. [file 12909_2026_9098_MOESM3_ESM.pdf]

Beijing Tsinghua Changgung Hospital Ethics Committee

Ethics audit and approval documents

No.:25268-6-01

Project: Application Effect of Defect-Based Scenario Simulation Teaching Method in the Specialized Skills Training of Anesthesia Nurses

Trial launch: Paper Publication

Whether drug or devices are involved in clinical trials: ☐ yes , ☑ no

If involved: **Stage:** / **Sponsor:** /

Principal: Yanli Ma **Department:**Department of Anesthesiology

Type: ☑ Initial audit ☐ follow-up audit: / ☐reaudit application

Mode: ☑ rapid audit ☐meeting audit

Material:

1.Ethics application form; 2. Informed consent;3.Manuscript.

Audit opinion: Agree to publish

According to the National Health Commission-《Human beings biomedical research ethics audit method (2023)》, the State Drug Administration/ National Health Commission-《the quality control standard for clinical trials (2020)》&《the medical equipment quality control standard for clinical trials (2022)》, WMA《Helsinki declaration (2013)》and CIOMS《the human body biomedical research international moral guideline ethical principles》, by the ethics committee audit, the trial project of "**Application Effect of Defect-Based Scenario Simulation Teaching Method in the Specialized Skills Training of Anesthesia Nurses**" is approved to publish according to the approved research protocol, informed consent, etc.

Beijing Tsinghua Changgung Hospital Ethics Committee

Mar.18, 2025

Executive Secretary of Ethics Committee: Manting Liu Tel: 56118567 Email: IRB@btch.edu.cn
Add: 168 Litang Road, Changping District, Beijing, 102218
