# Supplementary material for: Defect-based scenario simulation teaching in the specialized skills training of nurse anesthetists: a before–after within-subject design
Source: BMC Med Educ. 2026 Apr 1;26:752. doi: 10.1186/s12909-026-09098-7 (PMC13169835; doi:10.1186/s12909-026-09098-7)
Supplement: Supplementary file 4 — Supplementary Material 4. [file 12909_2026_9098_MOESM4_ESM.docx]

1. **Structured Checklists for Practical Skills Assessment**

Structured Assessment Checklist for Arterial Catheterization

Scoring Protocol:

Scoring Method: Binary scoring. 1 point = Standard Met; 0 points = Standard Not Met.

Final Score Calculation: Total Points Earned / Total Possible Points × 100.

| Dimension | Item | Assessment Criteria (Standard Operating Procedures) | Score (0/1) |
| --- | --- | --- | --- |
| I. Procedural Technique *(Weight: ~70%)* | 1 | Equipment Preparation: Prepare pressure kit, pressure bag, 500ml N.S., heparin, needles, sterile items. Ensure valid expiry dates and function. |  |
|  | 2 | Heparin Saline: Add heparin (1250IU) to 500ml N.S.; Label bag "ABP" & "Heparin". |  |
|  | 3 | System Priming: Tighten connections. Prime tubing with saline to exhaust all air (including stopcocks). |  |
|  | 4 | Pressurization: Place saline in pressure bag and inflate to >300 mmHg. |  |
|  | 5 | Zeroing: Mount transducer at phlebostatic axis (mid-axillary line). Perform zeroing sequence correctly (Off to patient -> Zero -> Off to air). |  |
|  | 6 | Positioning: Place wrist pad to extend wrist. Palpate artery to determine depth/direction. |  |
|  | 7 | Disinfection: Disinfect skin (10cm x 10cm), wait 30s to dry. Maintain sterile field. |  |
|  | 8 | Puncture: Perform successful puncture; connect tubing immediately; check waveform. |  |
|  | 9 | Check: Verify blood return, ensure no bubbles, re-verify zero point. |  |
|  | 10 | Fixation: Clean area. Secure catheter with dressing/tape (do not cover puncture site excessively). |  |
|  | 11 | Documentation: Record date and time on the dressing. |  |
|  | 12 | Waste Disposal: Dispose of sharps and waste in correct bins (Yellow/Black). |  |
|  | 13 | Indications: Correctly state at least 3 indications (e.g., hypotension anesthesia, major surgery, elderly). |  |
|  | 14 | Complications: Correctly state at least 3 complications (e.g., infection, thrombosis, spasm). |  |
|  | 15 | Management: Explain handling of vasospasm (no meds via line) and waveform errors. |  |
|  | 16 | Post-care: Explain requirement for compression (>5min) and observing limb circulation. |  |
| II. Communication Skills *(Weight: ~10%)* | 17 | Identify & Explain: Verify patient identity. Explain the purpose/steps to the patient (if conscious) to obtain cooperation. |  |
|  | 18 | Interaction: Respond to patient queries professionally during the procedure. |  |
| III. Humanistic Care *(Weight: ~10%)* | 19 | Privacy: Unbutton sleeve gently; expose only necessary area to protect privacy. |  |
|  | 20 | Comfort: Evaluate need for local anesthesia. Ensure comfort with wrist pad. |  |
| IV. Health Education *(Weight: ~10%)* | 21 | Purpose & Prevention: Explain the purpose/benefits of the catheter; Instruct patient not to touch or pull the catheter to prevent infection or dislodgment. |  |
|  | 22 | Symptom Reporting: Instruct patient to immediately report any discomfort at the puncture site, such as itching, redness, swelling, heat, pain, or bleeding. |  |
| TOTAL |  | Total Possible Points: 22 | ___ / 22 |
| FINAL CALCULATION |  | (Total Score ÷ 22) × 100 | = ___ Points |

Structured Assessment Checklist for Endotracheal Intubation Assistance

Scoring Protocol:

Scoring Method: Binary scoring. 1 point = Standard Met; 0 points = Standard Not Met.

Final Score Calculation: Total Points Earned / Total Possible Points × 100.

| Dimension | Item | Assessment Criteria (Standard Operating Procedures) | Score (0/1) |
| --- | --- | --- | --- |
| I. Procedural Technique *(Weight: ~70%)* | 1 | Asepsis & Self-Prep: Performs hand hygiene; Wears mask/gloves correctly. |  |
|  | 2 | Equipment Check: Checks anesthesia machine, suction, and laryngoscope light; Ensures circuit connections are tight. |  |
|  | 3 | Tube Cuff Test: Inflates cuff to test for leaks, then completely deflates it; Checks inflation line. |  |
|  | 4 | Connector Tightening (Critical): Specially Emphasized: Must tighten the respiratory circuit connector into the endotracheal tube firmly. |  |
|  | 5 | Lubrication & Stylet: Lubricates distal end; Inserts stylet (tip 1cm proximal to end); Shapes tube into "J". |  |
|  | 6 | Equipment Layout: Places laryngoscope, tape, syringe, tube, and stethoscope in an easily accessible position. |  |
|  | 7 | Dental Assessment: Checks for loose teeth before induction; Secures loose teeth if present. |  |
|  | 8 | Patient Positioning (New): Assists in positioning the patient's head (e.g., placing pillow) to achieve the optimal "Sniffing Position" for intubation. |  |
|  | 9 | Pre-Oxygenation: Administers oxygen effectively to the patient before induction. |  |
|  | 10 | Drug Administration: Administers anesthetic drugs strictly according to orders. |  |
|  | 11 | Assisting Intubation: Hands laryngoscope (Left) and tube (Right) smoothly; Applies cricoid pressure if requested. |  |
|  | 12 | Stylet Removal: Holds tube firmly; Smoothly removes stylet without obstructing operator's vision. |  |
|  | 13 | Inflation & Connection: Immediately inflates cuff (5-8ml); Connects tube to ventilator circuit. |  |
|  | 14 | Verification: Auscultates bilateral lungs and observes EtCO2 waveform to confirm placement. |  |
|  | 15 | Fixation: Adjusts tube depth; Secures tube firmly with adhesive tape. |  |
|  | 16 | Laryngoscope Care: Removes blade immediately; Places in tray for disinfection (Do not discard). |  |
|  | 17 | Indications & Selection: Correctly states  ≥2 indications AND formula for tube size/depth. |  |
|  | 18 | Complications & Mgmt: Correctly identifies potential complications and management strategies. |  |
| II. Communication Skills *(Weight: ~10%)* | 19 | Patient Identification: Verifies patient identity (Name/ID) using two identifiers. |  |
|  | 20 | Team Time-Out (New): Verbally confirms patient, procedure, and site with the team immediately before induction (Safety Check). |  |
|  | 21 | Closed-Loop Communication: Maintains closed-loop communication regarding drug administration and step confirmation. |  |
| III. Humanistic Care *(Weight: ~10%)* | 22 | Continuous Explanation: Provides explanations to obtain informed consent and reduce anxiety. |  |
|  | 23 | Dignity & Comfort: Maintains patient dignity (privacy, grooming); Uses photos/music if appropriate. |  |
|  | 24 | Eye Protection (New): Gently tapes the patient's eyelids closed immediately after loss of consciousness to prevent corneal abrasion/drying. |  |
| IV. Health Education *(Knowledge Application) (Weight: ~10%)* | 25 | Pre-Intubation Education: Explains purpose, sensation, and cooperation points to patient/family. |  |
|  | 26 | Post-Extubation Guidance: Explains normal sensations (sore throat), breathing/coughing techniques, and oral hygiene. |  |
| TOTAL |  | Total Possible Points: 26 | ____ / 26 |
| FINAL CALCULATION |  | (Total Score ÷ 26) × 100 | = ___ Points |

Structured Assessment Checklist for Spinal Anesthesia Assistance

Scoring Protocol:

Scoring Method: Binary scoring. 1 point = Standard Met; 0 points = Standard Not Met.

Final Score Calculation: Total Points Earned / Total Possible Points × 100.

| Dimension | Item | Assessment Criteria (Standard Operating Procedures) | Score (0/1) |
| --- | --- | --- | --- |
| I. Procedural Technique *(Weight: ~70%)* | 1 | Asepsis & Prep: Performs hand hygiene; Wears surgical mask correctly; Checks emergency cart and supplies. |  |
|  | 2 | Supplies Check: Prepares spinal kit, iodine/alcohol, drugs, and syringe. Checks validity and package integrity of the kit before opening. |  |
|  | 3 | Patient Preparation: Connects ECG monitoring; Ensures IV line is patent; Administers oxygen via mask/cannula. |  |
|  | 4 | Pre-procedure Assessment (Knowledge): Correctly states indications (e.g., lower limb surgery) AND at least 4 contraindications (e.g., hypovolemia, infection, coagulopathy, ICP, back injury). |  |
|  | 5 | Positioning (Critical): Assists patient into lateral decubitus position; Head on pillow; Knees flexed to chest; Back arched outward; Shoulders and hips perpendicular to the floor; Back aligned with bed edge. |  |
|  | 6 | Disinfection Assist: Pours antiseptic solution for the doctor without contaminating the sterile field. |  |
|  | 7 | Sterile Medication Assist: Allows doctor to draw meds; Holds ampoule/vial correctly; Does not cross sterile field; Maintains distance >30cm above the table. |  |
|  | 8 | Medication Verification: Performs double-check of medication name, concentration, and expiry with the doctor before drawing. |  |
|  | 9 | Intra-procedure Support: Maintains patient position (holds neck/knees firmly but gently) to prevent movement during puncture. |  |
|  | 10 | Vital Signs Monitoring: Closely observes BP, HR, and SpO2 during puncture; Reports abnormalities immediately. |  |
|  | 11 | Emergency Readiness (Total Spinal): Correctly states management for Total Spinal Anesthesia: Mask O2, Call for help, Assist with intubation/CPR. |  |
|  | 12 | Post-Anesthesia BP: Measures blood pressure immediately after anesthesia injection/position change. |  |
|  | 13 | Level Assessment: Assesses the sensory anesthesia plane (dermatome level) to ensure it meets surgical requirements. |  |
|  | 14 | Abnormality Mgmt: Recognizes signs of hypotension/dyspnea; Notifies doctor; Speeds up IV infusion or administers vasopressors as ordered. |  |
|  | 15 | Post-Procedure Positioning: Assists patient to return to supine position carefully after the block is fixed. |  |
|  | 16 | Waste Disposal: Disposes of sharps and waste strictly according to medical waste classification (Yellow/Black bins). |  |
| II. Communication Skills *(Weight: ~10%)* | 17 | Safety Check (Time-Out): Performs "Three-Way Verification" (Patient, Doctor, Nurse) to confirm patient identity, site, and procedure before starting. |  |
|  | 18 | Positioning Instruction: Explains the specific posture requirements to the patient clearly (e.g., "Curl up like a shrimp") to ensure safety and success. |  |
| III. Humanistic Care *(Weight: ~10%)* | 19 | Comfort & Pain Mgmt: Assesses pain tolerance during positioning; Moves patient gently; Uses pillows for support. |  |
|  | 20 | Privacy & Warmth: Minimizes body exposure (exposes only puncture site); Covers patient with blanket to maintain body temperature. |  |
| IV. Health Education *(Weight: ~10%)* | 21 | Post-Op Guidance: Informs patient about bed rest requirements (e.g., lying flat for 6 hours) and instructions for keeping the puncture site dry/clean. |  |
|  | 22 | Complication Recognition: Instructs patient/family to report warning signs immediately, such as severe headache, neurological symptoms (numbness/weakness), or signs of infection (fever/redness). |  |
| TOTAL |  | Total Possible Points: 22 | ___ / 22 |
| FINAL CALCULATION |  | (Total Score ÷ 22) × 100 | = ___ Points |

**B-The measuring tools used in the research**

**Sample Items of the Chinese Version of CCTDI (CCTDI-CV)**

Note: The CCTDI is a copyrighted instrument. To comply with copyright regulations, only representative sample items for each subscale are presented below, rather than the full scale.

| Subscale | Description (Translated from Chinese) | Sample Item (Translated from Chinese) |
| --- | --- | --- |
| 1. Truth-seeking | The habit of desiring the best possible understanding of any given situation; it is following reasons and evidence where ever they may lead, even if they lead one to question cherished beliefs | "I always look for evidence to support my opinion, even if it contradicts my beliefs." |
| 2. Open-mindedness | The tendency to allow others to voice views with which one may not agree. Open-minded people act with tolerance toward the opinions of others | "I am satisfied with my ability to understand other people’s viewpoints." |
| 3. Analyticity | The tendency to be alert to what happens next. This is the habit of striving to anticipate both the good and the bad potential consequences or outcomes | "Before making an important decision, I try to gather as much relevant information as possible." |
| 4. Systematicity | The tendency or habit of striving to approach problems in a disciplined, orderly, and systematic way | "I am good at developing a systematic plan to solve complex problems." |
| 5. Critical Thinking Self-confidence | The tendency to trust the use of reason and reflective thinking to solve problems | "I appreciate my ability to think precisely." |
| 6. Inquisitiveness | The tendency to be intellectually curious and eager to acquire new knowledge | "When faced with an important decision, I make an effort to collect all relevant information beforehand." |
| 7. Cognitive Maturity | Being prudent in making judgments, willing to withhold or revise decisions when appropriate, and open to multiple approaches to problem-solving. Recognizing that decisions may sometimes be necessary even in the absence of complete information. | "I often reflect on what is right and wrong in my practice and experiences." |

**Teaching Satisfaction Questionnaire**

Please evaluate the teaching model of the core skills training and fill in your responses truthfully.

| **Items** | **Strongly disagree** **strongly agree** | | | | |
| --- | --- | --- | --- | --- | --- |
| I believe the current teaching activities are reasonably organized (including time, location, etc.)? | 1 | 2 | 3 | 4 | 5 |
| I am satisfied with the teaching instructor’s guidance level? |  |  |  |  |  |
| I believe the current skills training helps reinforce relevant knowledge retention? |  |  |  |  |  |
| I believe the current skills training helps clarify my operational shortcomings? |  |  |  |  |  |
| I believe the current skills training reduces my error rate in clinical operations? |  |  |  |  |  |
| I believe the current skills training improves my speed in clinical operations? |  |  |  |  |  |
| I believe the current skills training enhances my emergency response ability in clinical work? |  |  |  |  |  |
| I believe the current teaching activities help improve nurses' comprehensive abilities? |  |  |  |  |  |
| I believe the defect-based scenario simulation teaching is meaningful? |  |  |  |  |  |
| I believe the design of this defect-based scenario simulation teaching is reasonable? |  |  |  |  |  |

C-Clinical Checklist for Nursing Core Skills

**Clinical Checklist for Arterial Catheterization**

Date:  Persons Checked:  Checks Completed: Inspector:

| Category | Frequency Count (Tally marks) | Category | Frequency Count (Tally marks) |
| --- | --- | --- | --- |
| Failure to verify patient identity |  | Use of Expired Sterile Supplies/Items |  |
| Failure to follow medical orders |  | Inaccurate Arterial Pulse Palpation/Localization |  |
| Failure to assess puncture sites and procedure-related contraindications |  | Hematoma due to Multiple Attempts/Technical Difficulty |  |
| Failure to assess arterial suitability (via Allen test) |  | Prohibited Maneuver: Return the guide wire |  |
| Incomplete Preparation of Equipment/Supplies |  | Unsuccessful Cannulation Attempt |  |
| Inadequate Pressure Bag Maintenance (<300 mmHg) |  | Improper handling of sharp instruments |  |
| Inadequate Connection of Arterial Pressure Sensor |  | Retained Item in Patient Bed/Area |  |
| Inadequate De-airing / Incomplete Air Removal from Arterial Pressure Line |  | Failure to Open/Release the Anti-Reflux Clamp |  |
| Failure to provide procedure explanation and obtain informed consent |  | Puncture Site Occluded by Dressing/Film |  |
| Failure to expose the puncture site |  | Date/Time of Arterial Catheter Insertion Not Documented |  |
| Improper Limb Positioning for Puncture |  | Catheter Dislodgement |  |
| Inadequate Limb Immobilization |  | Incorrect Transducer Zeroing |  |
| Failure to Adhere to Hand Hygiene Protocol |  | Failure to Zero the Pressure Transducer/Monitor |  |
| Inadequate Disinfection Area |  | Failure to Recognize Arterial Pressure Waveform Dampening/Distortion |  |
| Improper Disinfection Technique |  | Failure to Address/Correct Arterial Waveform Distortion |  |
| Contamination of Sterile/Prepped Field |  | Catheter/Line Occlusion |  |

Clinical Checklist for Endotracheal Intubation Assistance

Date:  Persons Checked:  Checks Completed: Inspector:

| Category | Frequency Count (Tally marks) | Category | Frequency Count (Tally marks) |
| --- | --- | --- | --- |
| Failure to verify patient identity |  | Endotracheal Tube Not Shaped with Stylet |  |
| Dental Assessment: Not Performed |  | Tube Tip Lubrication: Not Applied |  |
| Airway Assessment: Not Performed |  | Unsafe Advancement of Stylet (beyond the Murphy eye) |  |
| Failure to Notify Anesthesiologist of Loose Tooth |  | Improper Stylet Removal Technique (excessive speed) |  |
| Incomplete Preparation of Equipment/Supplies |  | Failure to Maintain a Clear Line of Sight During Stylet Removal |  |
| Video Laryngoscope Light Source (Insufficient/Broken) |  | Failure to Connect to Ventilator/Breathing Circuit Post-Intubation |  |
| Incorrect Endotracheal Tube Selection (size/type) |  | Failure to Verify Endotracheal Tube Placement Depth |  |
| Suction Device: Not Available |  | Failure to Auscultate Bilaterally for Breath Sounds |  |
| Failure to Perform Pre-operative Anesthesia Machine Check |  | Failure to Monitor End-Tidal Carbon Dioxide (ETCO₂) |  |
| Pre-induction Preparation Omissions:  Failure to confirm endotracheal tube size  Endotracheal tube not unpacked and ready for use |  | Inadequate Endotracheal Tube Securement |  |
| Use of Expired Sterile Supplies/Items |  | Unplanned Endotracheal Tube Displacement/Migration After Securement |  |
| Cuff Leak Test: Not Performed |  |  |  |

Clinical Checklist for Spinal Anesthesia Assistance

Date:  Persons Checked:  Checks Completed: Inspector:

| Category | Frequency Count (Tally marks) | Category | Frequency Count (Tally marks) |
| --- | --- | --- | --- |
| Failure to verify patient identity |  | Incomplete Preparation of Equipment/Supplies |  |
| Venous Access: Not Established |  | Failure to Adhere to Hand Hygiene Protocol |  |
| ECG Monitoring: Not Applied |  | Violation of Aseptic Technique: Breaching/Crossing the Sterile Field |  |
| Pre-procedure Oxygen: Not Administered |  | Aseptic Technique Violation Resulting in Field Contamination |  |
| Failure to provide procedure explanation and obtain informed consent |  | Breakdown in Team Safety Check During Medication Retrieval: Independent Verification Omitted |  |
| Incorrect Positioning for Spinal Anesthesia |  | Failure to Protect Patient Privacy |  |
| Patient Restraint: Not Assessed |  | Failure to instantly commence blood pressure monitoring and active management following successful puncture. |  |
| Failure to Provide Pre-procedural Instructions for Spinal Anesthesia |  | Failure to Assess/Test the Sensory Block Level (Anesthetic Plane) |  |
| Failure to Check Expiry Dates of Sterile Supplies |  | Improper Technique for Sensory Block Level Assessment |  |
