# Supplementary material for: Defect-based scenario simulation teaching in the specialized skills training of nurse anesthetists: a before–after within-subject design
Source: BMC Med Educ. 2026 Apr 1;26:752. doi: 10.1186/s12909-026-09098-7 (PMC13169835; doi:10.1186/s12909-026-09098-7)
Supplement: Supplementary file 5 — Supplementary Material 5. [file 12909_2026_9098_MOESM5_ESM.docx]

***Error rates in clinical practice quality audits***

| **Item** | **Problem points of verification** | **Occurrence count (error rate %)** | | **χ^2^ /*Fisher* test** |
| --- | --- | --- | --- | --- |
|  |  | **TT** | **DBSS** |  |
| Arterial catheterization | 1. Allen test not performed | 259 (89) | 76 (26) | <0.001 |
|  | 2. Inadequate disinfection area | 135 (46) | 11 (4) | <0.001 |
|  | 3. Inadequate limb immobilization | 87 (30) | 10 (3) | <0.001 |
|  | 4.Unsuccessful Cannulation Attempt | 83 (29) | 21 (7) | <0.001 |
|  | 5.Incomplete Air Removal from Arterial Pressure Line | 22 (8) | 4 (1) | <0.01 |
|  | 6.Return the guide wire | 22 (8) | 3 (1) | <0.001 |
|  | 7.Failure to Zero the Pressure Transducer/Monitor | 14 (5) | 4 (1) | <0.05 |
|  | 8.Incomplete Preparation of Equipment/Supplies | 15 (5) | 2 (0) | <0.01 |
|  | 9.Inaccurate Arterial Pulse Palpation/Localization | 11 (4) | 4 (1) | **>0.05** |
|  | 10.Inadequate Limb Immobilization | 9 (3) | 2 (0) | <0.05 |
|  | 11.Failure to expose the puncture site | 9 (3) | 1 (0) | <0.05 |
|  | 12.Retained Item in Patient Bed/Area | 5 (2) | 1 (0) | **>0.05** |
| Endotracheal intubation assistance | 1. Failure to monitor end-tidal carbon dioxide (ETCO_2_) | 178 (78) | 13 (6) | <0.001 |
|  | 2. Pre-induction preparation omissions:  Failure to confirm endotracheal tube size  Endotracheal tube not unpacked and ready for use | 132 (58) | 19 (8) | <0.001 |
|  | 3. Airway assessment not performed | 125 (55) | 17 (7) | <0.001 |
|  | 4.Dental Assessment: Not Performed | 119 (52) | 8 (3) | <0.001 |
|  | 5.Suction Device: Not Available | 101 (44) | 6 (3) | <0.001 |
|  | 6.Incomplete Preparation of Equipment/Supplies | 41 (18) | 5 (2) | <0.001 |
|  | 7.Failure to Connect to Ventilator/Breathing Circuit Post-Intubation | 24 (10) | 2 (1) | <0.001 |
|  | 8.Improper Stylet Removal Technique (excessive speed | 11(5) | 2(1) | <0.05 |
|  | 9.Inadequate Endotracheal Tube Securement | 4 (2) | 4 (2) | **>0.05** |
| Spinal anesthesia assistance | 1. Failure to check expiry dates of sterile supplies | 24 (25) | 7 (7) | <0.01 |
|  | 2. Failure to instantly commence blood pressure monitoring and active management following successful puncture | 17 (18) | 7 (7) | <0.05 |
|  | 3. Improper technique for sensory block level assessment | 16 (17) | 4 (2) | <0.01 |
|  | 1. Pre-procedure Oxygen: Not Administered | 11 (11) | 2 (2) | <0.001 |
|  | 5.Failure to Protect Patient Privacy | 11 (11) | 2 (2) | <0.001 |
|  | 6.Incorrect Positioning for Spinal Anesthesia | 9 (9) | 4 (4) | **>0.05** |
|  | 7.Patient Restraint: Not Assessed | 6 (6) | 1 (1) | **>0.05** |
|  | 8.Violation of Aseptic Technique: Breaching/Crossing the Sterile Field | 3 (3) | 0 (0) | **>0.05** |
