# Supplementary material for: Defect-based scenario simulation teaching in the specialized skills training of nurse anesthetists: a before–after within-subject design
Source: BMC Med Educ. 2026 Apr 1;26:752. doi: 10.1186/s12909-026-09098-7 (PMC13169835; doi:10.1186/s12909-026-09098-7)
Supplement: Supplementary file 7 — Supplementary Material 7. [file 12909_2026_9098_MOESM7_ESM.pdf]

Error rates in clinical practice quality audits

Item	Problem points of verification	Occurrence count (error rate %) TT	DBSS	χ^2 /*Fisher* test
Arterial catheterization	1. Allen test not performed	259 (89)	76 (26)	<0.001
	2. Inadequate disinfection area	135 (46)	11 (4)	<0.001
	3. Inadequate limb immobilization	87 (30)	10 (3)	<0.001
	4.Unsuccessful Cannulation Attempt	83 (29)	21 (7)	<0.001
	5.Incomplete Air Removal from Arterial Pressure Line	22 (8)	4 (1)	<0.01
	6.Return the guide wire	22 (8)	3 (1)	<0.001
	7.Failure to Zero the Pressure Transducer/Monitor	14 (5)	4 (1)	<0.05
	8.Incomplete Preparation of Equipment/Supplies	15 (5)	2 (0)	<0.01
	9.Inaccurate Arterial Pulse Palpation/Localization	11 (4)	4 (1)	**>0.05**
	10.Inadequate Limb Immobilization	9 (3)	2 (0)	<0.05
	11.Failure to expose the puncture site	9 (3)	1 (0)	<0.05
	12.Retained Item in Patient Bed/Area	5 (2)	1 (0)	**>0.05**
Endotracheal intubation assistance	1. Failure to monitor end-tidal carbon dioxide ($ETCO_2$)	178 (78)	13 (6)	<0.001
	2. Pre-induction preparation omissions: Failure to confirm endotracheal tube size Endotracheal tube not unpacked and ready for use	132 (58)	19 (8)	<0.001
	3. Airway assessment not performed	125 (55)	17 (7)	<0.001
	4.Dental Assessment: Not Performed	119 (52)	8 (3)	<0.001
	5.Suction Device: Not Available	101 (44)	6 (3)	<0.001

	6.Incomplete Preparation of Equipment/Supplies	41 (18)	5 (2)	<0.001
	7.Failure to Connect to Ventilator/Breathing Circuit Post-Intubation	24 (10)	2 (1)	<0.001
	8.Improper Stylet Removal Technique (excessive speed	11(5)	2(1)	<0.05
	9.Inadequate Endotracheal Tube Securement	4 (2)	4 (2)	**>0.05**
Spinal anesthesia assistance	1. Failure to check expiry dates of sterile supplies	24 (25)	7 (7)	<0.01
	2. Failure to instantly commence blood pressure monitoring and active management following successful puncture	17 (18)	7 (7)	<0.05
	3. Improper technique for sensory block level assessment	16 (17)	4 (2)	<0.01
	4. Pre-procedure Oxygen: Not Administered	11 (11)	2 (2)	<0.001
	5.Failure to Protect Patient Privacy	11 (11)	2 (2)	<0.001
	6.Incorrect Positioning for Spinal Anesthesia	9 (9)	4 (4)	**>0.05**
	7.Patient Restraint: Not Assessed	6 (6)	1 (1)	**>0.05**
	8.Violation of Aseptic Technique: Breaching/Crossing the Sterile Field	3 (3)	0 (0)	**>0.05**
